# Chemotherapy-triggered changes in stromal compartment drive tumor invasiveness and progression of breast cancer

**DOI:** 10.1186/s13046-021-02087-2

**Published:** 2021-09-27

**Authors:** Jana Plava, Monika Burikova, Marina Cihova, Lenka Trnkova, Bozena Smolkova, Pavel Babal, Lucia Krivosikova, Pavol Janega, Lucia Rojikova, Slavka Drahosova, Martin Bohac, Lubos Danisovic, Lucia Kucerova, Svetlana Miklikova

**Affiliations:** 1 Biomedical Research Center of the Slovak Academy of Sciences, Dubravska cesta 9, 845 05 Bratislava, Slovakia; 2 Department of Pathology, Faculty of Medicine, Comenius University in Bratislava, Sasinkova 4, 811 08 Bratislava, Slovakia; 3 Hermes LabSystems, s.r.o., Puchovska 12, 831 06 Bratislava, Slovakia; 4 2nd Department of Oncology, Faculty of Medicine, Comenius University, National Cancer Institute, Klenova 1, 833 10 Bratislava, Slovakia; 5 Department of Oncosurgery, National Cancer Institute, Klenova 1, Bratislava, Slovakia; 6 Regenmed Ltd, Medena 29, 811 08 Bratislava, Slovakia; 7 Institute of Medical Biology, Genetics and Clinical Genetics, Faculty of Medicine, Comenius University in Bratislava, Sasinkova 4, 811 08 Bratislava, Slovakia

**Keywords:** Breast cancer, Mesenchymal stromal cells, Chemotherapy, Tumor microenvironment, Cancer progression

## Abstract

**Background:**

Chemotherapy remains a standard treatment option for breast cancer despite its toxic effects to normal tissues. However, the long-lasting effects of chemotherapy on non-malignant cells may influence tumor cell behavior and response to treatment. Here, we have analyzed the effects of doxorubicin (DOX) and paclitaxel (PAC), commonly used chemotherapeutic agents, on the survival and cellular functions of mesenchymal stromal cells (MSC), which comprise an important part of breast tumor microenvironment.

**Methods:**

Chemotherapy-exposed MSC (DOX-MSC, PAC-MSC) were co-cultured with three breast cancer cell (BCC) lines differing in molecular characteristics to study chemotherapy-triggered changes in stromal compartment of the breast tissue and its relevance to tumor progression *in vitro* and *in vivo*. Conditioned media from co-cultured cells were used to determine the cytokine content. Mixture of BCC and exposed or unexposed MSC were subcutaneously injected into the immunodeficient SCID/Beige mice to analyze invasion into the surrounding tissue and possible metastases. The same mixtures of cells were applied on the chorioallantoic membrane to study angiogenic potential.

**Results:**

Therapy-educated MSC differed in cytokine production compared to un-exposed MSC and influenced proliferation and secretory phenotype of tumor cells in co-culture. Histochemical tumor xenograft analysis revealed increased invasive potential of tumor cells co-injected with DOX-MSC or PAC-MSC and also the presence of nerve fiber infiltration in tumors. Chemotherapy-exposed MSC have also influenced angiogenic potential in the model of chorioallantoic membrane.

**Conclusions:**

Data presented in this study suggest that neoadjuvant chemotherapy could possibly alter otherwise healthy stroma in breast tissue into a hostile tumor-promoting and metastasis favoring niche. Understanding of the tumor microenvironment and its complex net of signals brings us closer to the ability to recognize the mechanisms that prevent failure of standard therapy and accomplish the curative purpose.

**Supplementary Information:**

The online version contains supplementary material available at 10.1186/s13046-021-02087-2.

## Background

Breast cancer is generally referred to as the leading cancer type in women [1] and even despite the constant advances in cancer biology knowledge and cancer treatment, breast cancer therapy still meets several challenges. Current treatment options for breast cancer include mostly surgical approaches, radiotherapy, immunotherapy, targeted therapy or chemotherapy. Chemotherapeutic agents mediate their effects through various modes of action that eventually target distinct aspects of tumor cell metabolism [2]. Widely used drugs include alkylating agents and platinum-based compounds which are involved in formation of DNA crosslinks leading to the impairment of DNA replication, while anti-metabolites target crucial cellular enzymes or act as analogs of nucleosides [3, 4]. Topoisomerase inhibitors interfere with the activities of DNA topoisomerases I and II which are involved in DNA strand cleavage and thus disrupt DNA replication [5]. Furthermore, antineoplastic antibiotics and anthracyclines like doxorubicin (DOX) and daunorubicin exert their functions via multiple mechanisms including inhibition of type II topoisomerases, intercalation within DNA or generation of reactive oxygen species [6, 7]. Lastly, vinca alkaloids and taxanes like paclitaxel (PAC) or docetaxel are encompassed in the group of microtubule inhibitors that affect polymerization and depolymerization of microtubules leading to the disruption of mitosis [8].

The purpose of the chemotherapy, neoadjuvant or adjuvant, is to eliminate disseminated tumor cells and provide recurrence-free and metastasis-free survival or to secure improved outcomes in breast cancer patients. The main goal of chemotherapy is to target fast-dividing cells, but other tissues in the body are not spared its cytotoxic effects either. The cells comprising the tumor microenvironment (TME) are exposed to the drugs equally and since they are in active communication with the tumor cells, the changes inflicted by the chemotherapy might affect the vivid cellular cross-talk in a manner not entirely known. However, it is now generally accepted that the bidirectional communication between tumor cells and the surrounding stromal components is capable of dictating the therapeutic response [9]. As documented in many research articles, tumor cells and stromal cells may interact in ways that either favor or hamper tumor progression. Under cytotoxic therapy, tumor evolution becomes inevitable and it is clear that there are multitude mechanisms by which the efficacy of cytotoxic agents can be reduced by cells within the TME [10]. Moreover, the cells comprising the breast TME, such as fibroblasts, pericytes, or mesenchymal stromal cells (MSC) can become activated in tumors [10] and may influence cancer progression and metastasis [11]. On top of that, the presence of the nervous system in tumors has also recently started to receive attention [12]. Tumor cells together with the surrounding reactive stroma release neurotrophins to recruit nerves necessary for their growth [13]. Stromal cells were also found to express receptors, which are targeted by the corresponding ligands secreted by nerves [14]. Through this reciprocal interaction, the nervous system is involved in cancer progression and metastasis, an aspect nicely reviewed recently, discussing the sympathetic nervous system as an important modulator of breast cancer metastasis [15].

MSC are key contributors of the breast tumor microenvironment, but their role in cancer progression is not fully understood. They can have positive, but also negative roles on breast cancer cells, which is important in determining therapeutic potential of these cells [16–18]. Herein, we examined the effect of chemotherapy exposure on MSC, a particularly abundant cell population within the breast adipose tissue, and evaluated their chemotherapy-induced activation in terms of potential pro-tumorigenic effect.

## Materials and methods

### Cell Cultures

Human mammary gland adenocarcinoma cell lines MDA-MB-231 (ATCC® HTB-26™, further as MDA), JIMT-1 (DSMZ no.: ACC 589, further as JIMT) and T-47D (ATCC® HTB-133™, further as T47D) were transduced with IncuCyte® NucLight Lentivirus Reagents (Essen BioScience, Ann Arbor, MI, USA) to express a nuclear red fluorescent protein (mKate2) for visualization in co-culture experiments - except for Gap-Junctional Intercellular Communication (GJIC) evaluation described below, where parental cells were used.

MSC were isolated from the breast adipose tissue of healthy women. Biopsy samples ranging from 3–10 cm^3^ were obtained during plastic surgeries. All donors provided informed consent and all procedures were approved by the Ethics Committee of the Ruzinov University Hospital. MSC were isolated by plastic adherence technique and subsequently characterized as previously described in Plava et al. [19], and used before reaching the 10th passage.

Breast cancer cell lines were cultured in high-glucose (4.5 g/L) Dulbecco’s modified Eagle medium (DMEM, PAA Laboratories GmbH, Pasching, Austria) supplemented with 10 % fetal bovine serum (FBS, Biochrom AG, Berlin, Germany), 2 mM glutamine (PAA Laboratories GmbH), 10 000 IU/mL penicillin (Biotica, Part. Lupca, Slovakia), 5 µg/mL streptomycin (PAA Laboratories GmbH) and 2.5 µg/mL amphotericin B (Sigma-Aldrich, Taufkirchen, Germany). After isolation, the MSC were maintained in low-glucose (1 g/L) DMEM (PAA Laboratories GmbH) supplemented with the same concentration of serum and antibiotics. All cells were maintained at 37 °C in a humidified atmosphere and 5 % CO_2_. If stated, MSC were exposed to DOX 50 ng/ml (DOX-MSC) or PAC 4 ng/ml (PAC-MSC) for 48 h.

### Drug Sensitivity Assay

To evaluate the chemosensitivity to DOX and PAC, MSC were seeded in 96-well plates in triplicates (5 × 10^3^ MSC/well). Twenty-four hours later, the cells were exposed to DOX (50 ng/ml; Sigma-Aldrich) or PAC (4 ng/ml; Selleck Chemicals, Houston, USA) and cultured for 48 h. Concentrations were selected based on IC_50_ values of breast cancer cells. Cell viability after exposure was measured using CellTiter-Glo® Luminescent Cell Viability Assay (Promega Corporation, Madison, WI, USA) according to the manufacturer´s protocol and measured on GloMax® Discover System (Promega Corporation). Values were expressed as means of replicates ± SD. Luminescence of unexposed cells served as a reference. Experiments were repeated 3x and representative results are shown.

### Cytokines Determination

Secretion of human cytokines and chemokines was analyzed using the Proteome Profiler Human XL Cytokine Array Kit (for determination of MSC cytokines) or Proteome Profiler Human Cytokine Array Kit (for co-culture experiments) (both R&D Systems™, Minneapolis, MN, USA). MSC were exposed to the DOX 50 ng/ml or PAC 4 ng/ml for 48 h. Serum-free conditioned medium harvested from 2 × 10^5^ MSC (unexposed, DOX-MSC, PAC-MSC), or in case of co-culture experiments from 2 × 10^5^ JIMT/MDA/T47D + 1 × 10^5^ MSC after 2 days and subsequently loaded on the membranes spotted with cytokine antibodies. Membranes were scanned on Li-Cor Oddysey Fc Imaging System (Li-Cor Corporation, USA) and quantitatively evaluated using ImageJ software (NIH, Bethesda, MD, USA).

### Enzyme-Linked Immunosorbent Assay (ELISA)

Protein levels of Dkk-1 were determined using Human Dkk-1 Quantikine ELISA Kit (R&D Systems™). A serum-free conditioned culture medium obtained from 1 × 10^5^ MSC (unexposed or exposed to DOX or PAC for 48 h) was used in the assay according to the manufacturer´s protocol. Absorbance was measured on the xMark™ Microplate Absorbance Spectrophotometer (Bio-Rad Laboratories Ltd, Watford, UK).

### Gap-Junctional Intercellular Communication

GJIC between breast tumor cells and MSC was detected by a fluorescent dye transfer as described previously [20]. Stained monolayer of MSC (unexposed or exposed to DOX or PAC for 48 h) was mixed in a 1:1 ratio with stained cancer cells and seeded on a 6-well plate (500,000 cells/well) for a 24-hour co-culture. Co-cultures were washed with PBS, detached with Accutase® (Sigma-Aldrich) and the dye transfer was measured on BD FACSCanto™ II Flow cytometer (Becton Dickinson, USA) equipped with the FacsDiva program. The fresh mix of MSC and tumor cells were used as a control of GJIC formation. Data were analyzed by FCS Express Flow Cytometry Software (De Novo Software, Pasadena, CA, USA).

### Evaluation of the chemotherapy-exposed MSC effect on cancer cell proliferation

Kinetic evaluation of the cancer cell proliferation in a co-culture with MSC (unexposed or exposed to DOX or PAC for 48 h)was based on the tumor cells’ red nuclei counting using IncuCyte ZOOM™ kinetic imaging system (Essen BioScience). Cells were seeded together in a 2:1 ratio in quadruplicates (2 × 10^3^ cancer cells + 1 × 10^3^ MSC) on a 96-well plate in standard culture medium. Each well was imaged every two hours until the cells reached confluence. The tumor cell number was evaluated by IncuCyte ZOOM™ software (Essen BioScience) based on the enumeration of tumor cells’ expressing nuclear red fluorescent protein by kinetic imaging scanning. The values are expressed as means of replicates ± SD.

### *In Vivo* Experiments

Six-week-old female SCID/Beige mice (Charles River, Germany) were used in accordance with institutional guidelines and approved protocols. The animals were bilaterally subcutaneously injected with a mixture of 5 × 10^5^ MSC (unexposed, DOX-MSC, PAC-MSC) and 1 × 10^6^ MDA cells re-suspended in 100 µL serum-free DMEM diluted 1:1 with extracellular (ECM) gel (Sigma-Aldrich).

For both *in vivo* experiments, the animals were divided into four groups: (1) control group of MDA alone (n = 5 in the first experiment, and n = 6 in the second one); (2) MDA co-injected with unexposed MSC (n = 4, n = 6); (3) MDA co-injected with DOX-MSC (*n* = 5, *n* = 6); and (4) MDA co-injected with PAC-MSC (n = 5, n = 6). The animals were regularly inspected for tumor growth, and were sacrificed according to the ethical guidelines when the tumor volume exceeded 1 cm^3^. Throughout the experiment, the tumors were measured by a caliper, and tumor volume was calculated based on the following formula: tumor volume (mm^3^) = 0.52 × ((width + length)/2)^3^. The results were evaluated as the mean of tumor weight or tumor volume. The tumor weights were measured at the end of the experiment. The tumors were fresh frozen for molecular analysis or formalin-fixed for histological and immuno-histochemical analyses as described below.

To detect metastatic cells, excised lungs were evaluated for the red fluorescent signal emitted by MDA cells using an IVIS system (*In vivo* Imaging System, Caliper Life Sciences, MA, USA). Image sequences were acquired for two pairs of lungs from each tested group at 14 combinations of excitation and emission filters, at field of view B (FOV B). Spectral unmixing was performed to separate the specific fluorescent signal emitted by the fluorophore and tissue autofluorescence. All lungs were marked as separate Regions of Interest (ROIs), and the fluorescent signal was integrated over the whole ROI area and expressed as total radiant efficiency. This variable is normalized and allows the comparison between different images.

For quail chorioallantoic membrane (CAM) model fertilized Japanese quail (*Coturnix japonica*) eggs from a breeding colony (Laying Line 01, Institute of Animal Biochemistry and Genetics, Centre of Biosciences, Slovak Academy of Sciences) were processed as previously described in Debreova at al. [21]. The cells (1 × 10^6^ of tumor cells + 5 × 10^5^ unexposed/exposed MSC) were applied into a silicone ring (6 mm) placed on the CAM surface on embryonic day 7 (ED7). After 72 h, CAM images were obtained using Canon DS126291 camera. Afterward, the embryos were fixed with 4 % paraformaldehyde/2 % glutaraldehyde for 24 h at room temperature. The CAMs of fixed embryos were carefully dissected, mounted without folds onto glass slides and dried for analysis of angiogenesis.

Changes in blood vessels were quantified by the fractal analysis providing the fractal dimension (Df) estimate. The CAMs were illuminated using a transilluminator as a source of homogenous light and photographed (Canon DS126291). The subsequent image processing was performed as described in Vyboh et al. [22]. The fractal coefficient was analyzed with ImageJ software and calculated following the procedures described by Parsons-Wingerter et al. [23, 24].

### Histological and Immunohistochemical (IHC) Staining

Formalin-fixed, paraffin-embedded tumor tissues were cut into 5 µm sections, processed as described previously in Plava et al. [19] and double stained with anti-human Ki-67 MIB-1 and anti-human Vimentin (FLEX, DAKO, Carpinteria, CA, USA). Ki-67 positive staining was visualized by 3,3’- Diaminobenzidine (DAB substrate-chromogen solution, DAKO) for 5 min (brown color). Vimentin positive staining was visualized by Magenta (EnVision FLEX HRP Magenta Substrate Chromogen, DAKO) for 8 min (red color). Staining patterns were analyzed with Axio Scope A1, Zeiss microscope with Axiocam 105 color.

For analysis of neural infiltration, the tumor sections were stained with hematoxylin and eosin (HE) and immunohistochemically stained with two different polyclonal antibodies identifying nerve tissue; anti-S100 protein antibody, ready for use (DAKO) and anti-PGP9.5 protein antibody (Abcam, Cambridge, UK) in 1:200 dilution. For the detection of epithelial-to-mesenchymal transition (EMT), SLUG (Santa Cruz, A-7, Santa Cruz Biotechnology) diluted 1:50 in Dako REAL antibody diluent (DAKO) and anti-vimentin RTU antibody (DAKO). Macrophages were visualized using the anti-CD68 RTU antibody (DAKO) The immuno-reactivity was visualized with En-Vision kit (DAKO) and the peroxidase reaction product was developed with diamino benzidine (DAKO). The location and intensity of the nerve tissue markers S100 and PGP9.5 expressions were evaluated in light microscope Eclipse 80i (Nikon Corporation, Tokyo, Japan).

### Histological and morphometric evaluation

Infiltration extent of the tumor into surrounding adipose tissue was evaluated in the initial and the last tissue section after sectioning of slices for immunohistochemistry. The slides were stained with HE and the percentage with infiltrative interface of the whole tumor perimeter was morphometrically established (ImageJ 1.38).

Stained tumor and the adjacent adipose tissue nerve structures cross-section area up to 2 mm from the tumor surface was measured through two-dimensional picture analysis after slides digitization (ImageJ 1.38). The sum of the stained nerve areas was expressed as the percentage of the tumor section area in each experimental group.

Neutrophilic granulocytes were detected based on detection of enzymatic activity of chloracetate esterase (CHAE) with the substrate α-naphtol-AS-D-chloroacetate (Sigma-Aldrich).

### Gene Expression Arrays

To analyze the expression of 84 metastasis-related genes in mice tumors, RT² Profiler™ PCR Array Human Tumor Metastasis (PAHS-028ZD) was employed (Qiagen, Hilden, Germany). RNA was isolated by RNeasy® Lipid Tissue Mini Kit (Qiagen) and reverse transcription was performed using RT^2^ First Strand Kit (Qiagen). Quantitative real-time PCR was performed using SYBR® Green qPCR Mastermix (Qiagen) and Bio-Rad CFX96™ Touch Real-Time PCR Detection system (Bio-Rad Laboratories Ltd). The CT cut-off was set at 35, and targets expressed at very low quantities or undetected in the control group were excluded from further analysis. The ΔCt values for each group were averaged and used for fold-change calculations. Relative quantification of gene expression by ΔΔCt method was performed using expression profile of tumors from “MDA only” group as a control. Only genes whose expression exceeded 2.5-fold are shown in the graph.

### Statistical analysis

SPSS software package version 23 (IBM SPSS, Inc., Chicago, IL, US) and Graph Pad Prism 8.0.1. software (La Jolla, CA, USA) were used for statistical analysis. The normality of data distribution was assessed by the Shapiro-Wilk test. One-way analysis of variance (ANOVA) with Post-Hoc tests (Bonferroni or Dunnett T3 test) depending on assumed variances was used to compare differences in measured variables between exposed and control groups. Tukey’s multiple comparisons test was applied for analysis of IHC data. The effect of individual exposures on cell viability and tumor volume over several time points was evaluated using a general linear model for repeated measures. Statistical analysis of qPCR data was performed using relative expression software tool REST (REST 2009-RG Mode, Qiagen, Hilden, Germany) developed by Pfaffl et al. [25]. The p values < 0.05 were considered to be statistically significant.

## Results

### Chemotherapy-triggered changes in stromal compartment affect breast cancer cell behavior

Adipose tissue represents an important component of female breast and an accessible source of MSC, which are exposed to chemotherapeutic drugs together with tumor cells. MSC used in our experiments were exposed to DOX or PAC and subsequently co-cultured with three BC cell lines differing in molecular characteristics to study chemotherapy-related changes in stromal compartment of breast tissue and its relevance for tumor progression.

**Fig. 1:**
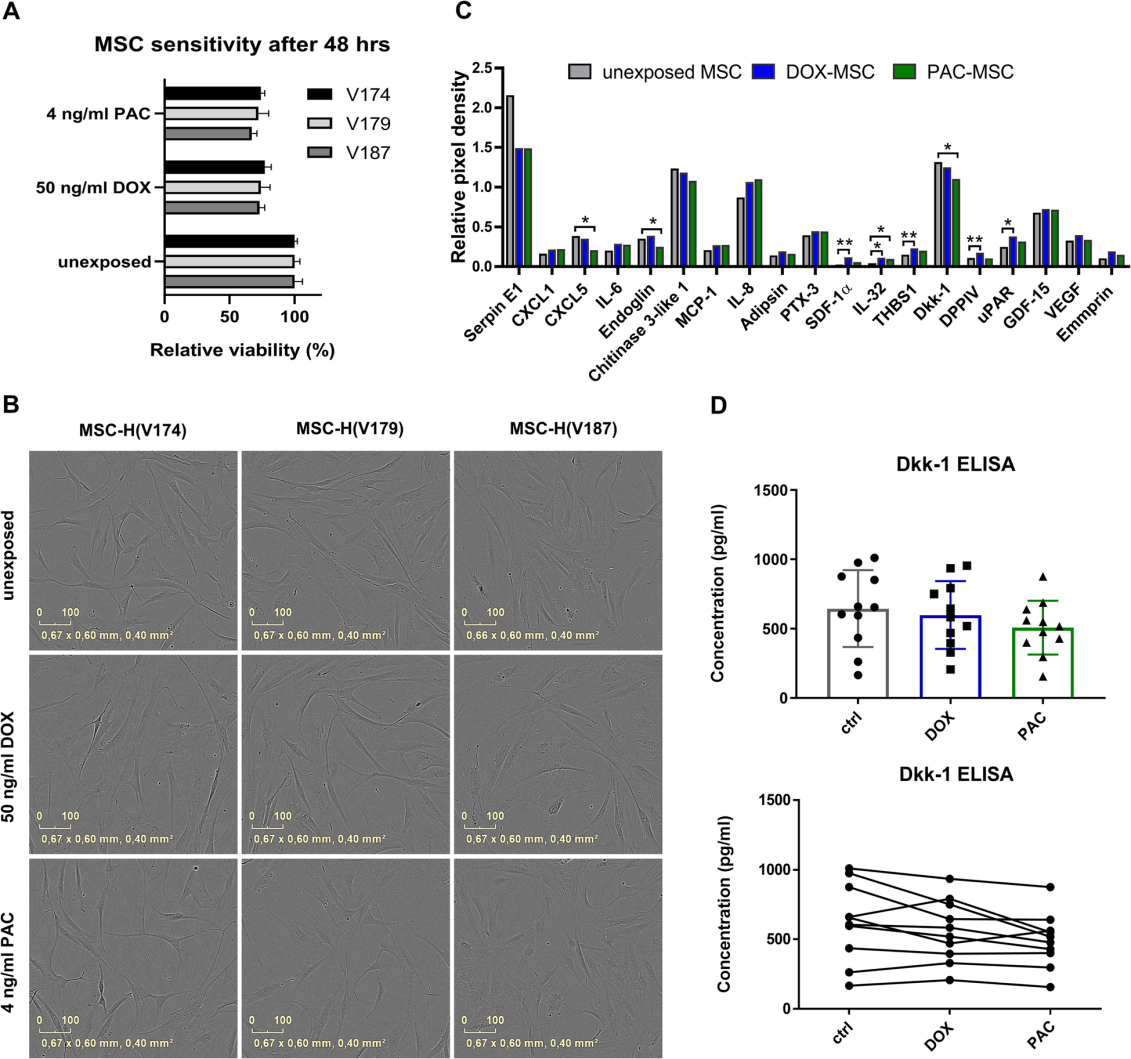
Chemotherapy-exposed MSC. (**A**) Chemosensitivity of three different isolates of MSC to doxorubicin (DOX, 50 ng/ml) and paclitaxel (PAC, 4 ng/ml) after 48-hour treatment. (**B**) Morphology of MSC exposed to chemotherapeutics. (**C**) Cytokine determination using the Proteome Profiler Human XL Cytokine Array Kit revealed that DOX exposure was associated with increased secretion of SDF-1α, IL-32, THBS1, DPPIV and uPAR in DOX-MSC compared to unexposed MSC. PAC exposure resulted in decreased production of CXCL5, endoglin, Dkk-1 and increased production of IL-32. (**D**) Exposure-induced differences in Dkk-1 levels were validated in various MSC isolates. Dunnett T3 test was used for statistical evaluation. Statistically significant results are highlighted with asterisks at *p* < 0.05 (*), *p* < 0.01 (**)

MSC were previously reported to be relatively resistant to chemotherapeutic drugs. We have shown that 48-hour exposure to DOX (50 ng/ml) or PAC (4 ng/ml) resulted in 25 % resp. 29 % decrease of viability on average (Fig. 1A). No marked changes in cell morphology in three different isolates of MSC were found (Fig. 1B). However, analysis of individual MSC secretory phenotype after the exposure revealed changes in production of pleiotropic cytokines, chemokines and growth factors (Fig. 1C). When we analyzed Dkk-1 levels in different MSC isolates by ELISA test, a broad range of concentrations even in unexposed MSC was observed suggesting isolate-specific cytokine levels (Fig. 1D).

**Fig. 2:**
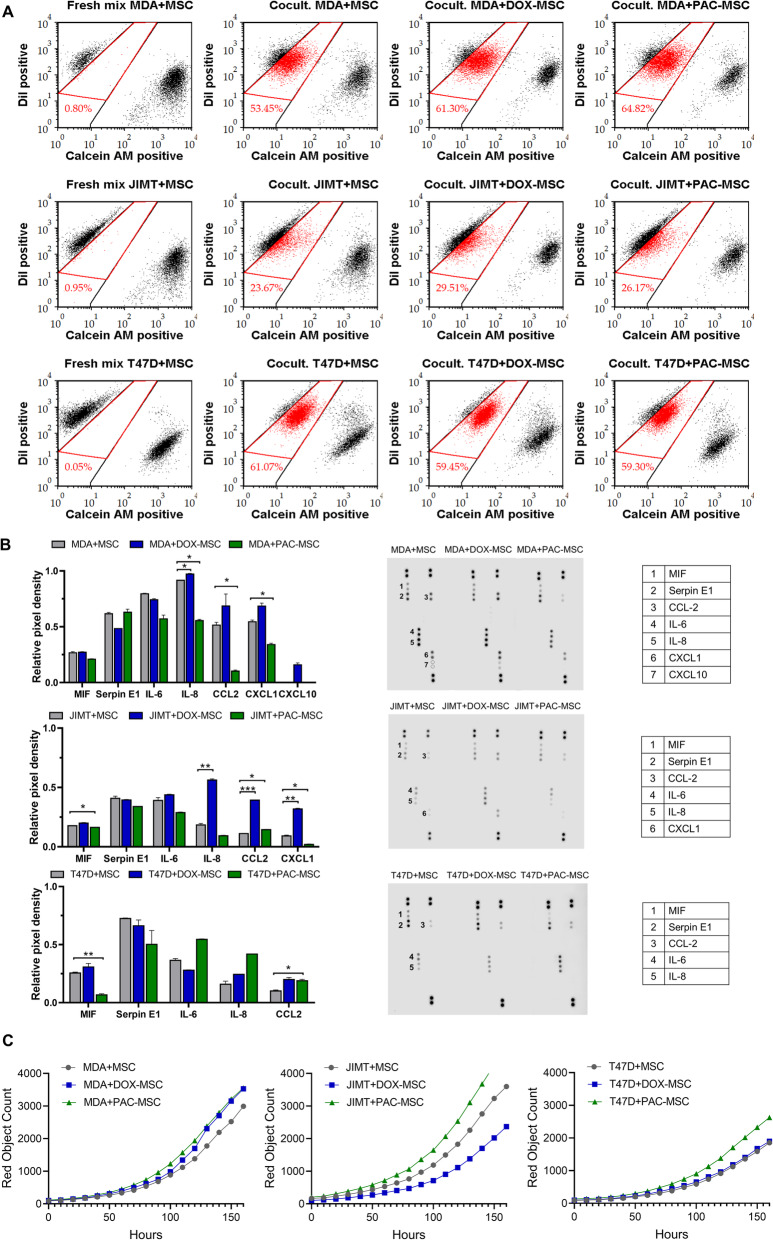
Direct co-culture of chemotherapy-exposed MSC and breast cancer cells. (**A**) Flow cytometry analysis of gap junctions showed higher percentage of double-positive MDA and JIMT cells (red) in co-culture with DOX-/PAC-MSC compared to unexposed MSC. (**B**) Cytokine secretion analysis in tumor cell-MSC co-culture. (**C**) The effect of chemotherapy-exposed MSC on tumor cells proliferation analyzed by IncuCyte ZOOM™ kinetic imaging system. For statistical evaluation, the one-way ANOVA with Turkey´s or the Dunnett T3 multiple comparison test was used, where the cytokine production in unexposed MSC or tumor cells mixed with unexposed MSC was set as a control. * *p* < 0.05, ** *p* < 0.01, *** *p* < 0.001

To assess the effect of chemotherapy-exposed MSC on communication with tumor cells, gap junctions were analyzed in their co-culture. Breast cancer cells were loaded with an impermeable lipophilic membrane dye DiI that stains the entire cell membrane while MSC were stained with a lipophilic cell membrane permeable dye, Calcein AM. Calcein AM is transferred to the DiI stained tumor cells through gap junctions. The assessment of this uptake was quantitated using flow cytometry where the fresh mix of MSC and BCC was used for gating strategy (Fig. 2A left). Chemotherapy-exposed MSC compared to unexposed MSC transferred the molecules more effectively. When co-cultured with MDA cells, the percentage of double-positive cells was 53.45 % (MDA + MSC), 61.30 % (MDA + DOX-MSC) and 64.82 % (MDA + PAC-MSC). In co-culture with JIMT cells, the percentage of double positive cells was 23.67 % (JIMT + MSC), 29.51 % (JIMT + DOX-MSC) and 26.17 % (JIMT + PAC-MSC). The analysis of gap junctions in T47D + MSC co-culture revealed only a small decrease in the percentage of double-positive cells (Fig. 2A right).

Cytokine production in co-culture of DOX-/PAC-MSC and BCC was analyzed in conditioned media harvested after 48 h. Co-culture of DOX-MSC with MDA or JIMT resulted in an increased level of IL-8, CCL2, and CXCL1 in the conditioned media, while the co-culture with PAC-MSC oppositely decreased their secretion (except for CCL2 which was increased when JIMT + PAC-MSC were co-cultured). In the co-culture of PAC-MSC with T47D, MIF secretion was decreased and CCL2 secretion was increased (Fig. 2B). The proliferation of NLR-stained breast cancer cells in the presence of MSC was evaluated by IncuCyte ZOOM™ software based on the enumeration of tumor cells’ expressing nuclear red fluorescent protein by kinetic imaging scanning every two hours. The impact of exposed MSC on proliferation was the most evident in JIMT cells co-cultured with DOX-MSC and PAC-MSC (Fig. 2C).

### Chemotherapy-exposed MSC drive invasive behavior of breast cancer cells *in vivo*

**Fig. 3:**
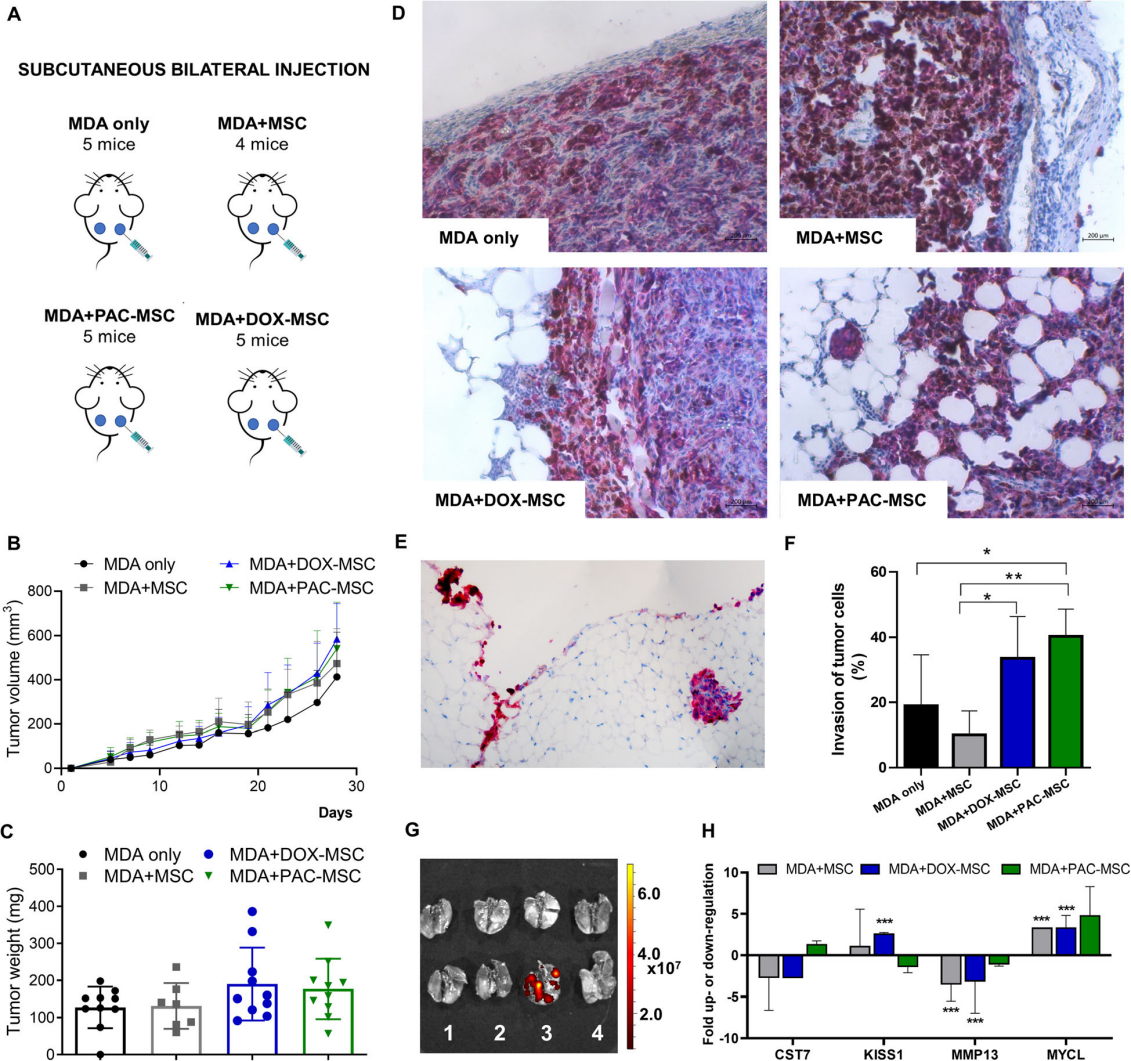
The effect of chemotherapy-exposed MSC on triple-negative breast cancer xenografts *in vivo*. (**A**) Scheme of experiment design. (**B**) Tumor volume evaluation. We observed faster tumor growth in groups where MSC were co-injected with cancer cells, but these changes were not statistically significant. (**C**) Tumor weights were measured after the experiment termination. We observed higher weight of tumors in groups containing DOX- and PAC-MSC, but these changes were not significant. (**D**) Representative pictures of Ki-67/VIM immunohistochemically stained xenografts’ periphery showed increased ability of tumor cells to invade into the surrounding stroma in xenografts formed by MDA cells co-injected with DOX- and PAC-MSC. (**E**) Increased metastatic potential of cancer cells in MDA + DOX-MSC group was additionally confirmed by VIM staining of metastatic cancer cells along the adipose tissue. (**F**) The percentage of the tumor perimeter with tumor cells infiltrative interface with the surrounding adipose tissue. Significantly higher tumor cell invasion was observed in groups, where MSC were exposed to DOX and PAC (MDA only vs. MDA + PAC-MSC, *p* = 0.038; MDA + MSC vs. MDA + PAC-MSC, *p* = 0.006; MDA + MSC vs. MDA + DOX-MSC, *p* = 0.042) compared to the group with unexposed MSC (**G**) Representative *ex vivo* image of lungs containing metastatic cells in group with DOX-MSC. Imaging was performed immediately after mice were sacrificed using IVIS Caliper. Two lungs from each group were used for imaging, metastases were detected by the red fluorescence of subcutaneously injected MDA cells. On the scale of fluorescence intensity, yellow color represents the strongest emission. 1 = MDA only, 2 = MDA + MSC, 3 = MDA + DOX-MSC, 4 = MDA + PAC-MSC. (**H**) RT² Profiler™ PCR Array Human Tumor Metastasis was used to assess gene expression changes in selected mice tumors. Only four out of 84 genes showed higher than 2.5-fold up- or down- regulation in MDA + unexposed or DOX-/PAC-exposed MSC tumors compared to MDA only group. Statistically significant results are highlighted with asterisks at *p* < 0.05 (*), *p* < 0.01 (**), *p* < 0.001 (***)

To evaluate the effect of chemotherapy-exposed MSC *in vivo*, MDA only or in the combination with unexposed/exposed MSC (MDA +MSC, MDA + DOXMSC, MDA + PAC-MSC) were subcutaneously bilaterally injected to six to eight-week-old female SCID beige mice (following the scheme in Fig. 3).

#### Immunodeficient SCID/Beige mice

The tumor volume and tumor weight analysis did not reveal supportive effect of chemotherapy-exposed MSC co-injected with tumor cells (Fig. 3B, C). Although differences between tumor size were not significant, the IHC analysis of tumor composition revealed marked changes at the tumor periphery. Representative Ki-67 and VIM staining of tumors composed solely of tumor cells showed lack of tumor cell migration at tumor periphery or only solitary migration of tumor cells co-injected with unexposed MSC. In contrast, histological analysis of tumors formed by breast cancer cells co-injected with chemotherapy-exposed MSC showed collective cell migration along adipocyte intercellular spaces (Fig. 3D). The increased invasive behavior of tumor cells was characterized by their invasion into the crown-like structures of adipocytes surrounding the tumors formed by MDA + DOX-MSC and MDA + PAC-MSC. The adipocytes closest to the tumor invasive front were smaller with wider intercellular spaces. The supportive effect of DOX-MSC on metastatic potential of tumor cells was documented also by the presence of VIM-positive metastatic tumor cells alongside the adipose tissue (Fig. 3E) and in the lungs of MDA + DOX-MSC mice (Fig. 3G). To quantify and statistically evaluate the tumor cells invasion potential, the percentage of the tumor perimeter encapsulations and the tumor infiltration of neighboring adipose tissue were evaluated (Fig. 3F). We observed significantly higher invasion of tumor cells in groups, where MSC were exposed to DOX and PAC (ANOVA, Bonferroni corrections) compared to the group with unexposed MSC, which provided an additional proof that chemotherapy exposure of MSC supported cancer cells invasion. Additional staining of tumor periphery was performed to analyze EMT using SLUG and VIM antibodies (Additional File 1 A, B). The differences between individual groups were not significant, but we noticed higher expression of SLUG in tumors, where MSC were co-injected with cancer cells compared to MDA only tumors. The SLUG protein was localized mostly on the cell membrane, but without significant differences between unexposed and DOX- or PAC-exposed groups. Moreover, macrophages and neutrophilic granulocytes were stained using anti-CD68 antibody and CHAE activity, respectively (Additional File 1 C, D). The presence of MSC led to increased density of neutrophils and macrophages, mostly at the periphery of the xenografts and the treatment with DOX or PAC led to additional increase in their density, especially in macrophages.

In the next step, an expression of 84 genes associated with metastatic potential of cancer cells was analyzed in selected tumors using the metastasis PCR arrays (Fig. 3H). Tumors in every group containing MSC were compared to the expression in tumors formed by MDA cells only. All three tested groups were characterized by downregulation of *MMP13*, a protein involved in the ECM breakdown, and upregulation of *MYCL*, which is associated with cancer susceptibility as well as metastasis, prognosis, and adverse survival. In the tumors formed by MDA + PAC-MSC compared to MDA only, a down-regulation of *KISS1*, the precursor of metastasis suppressor proteins, was observed. However, *CST7*, which was previously shown to be an important mediator within the TME affecting the cytotoxicity of NK cells and consequently antitumor immune response, was down-regulated in all groups except for the MDA + PAC-MSC tumors.

**Fig. 4:**
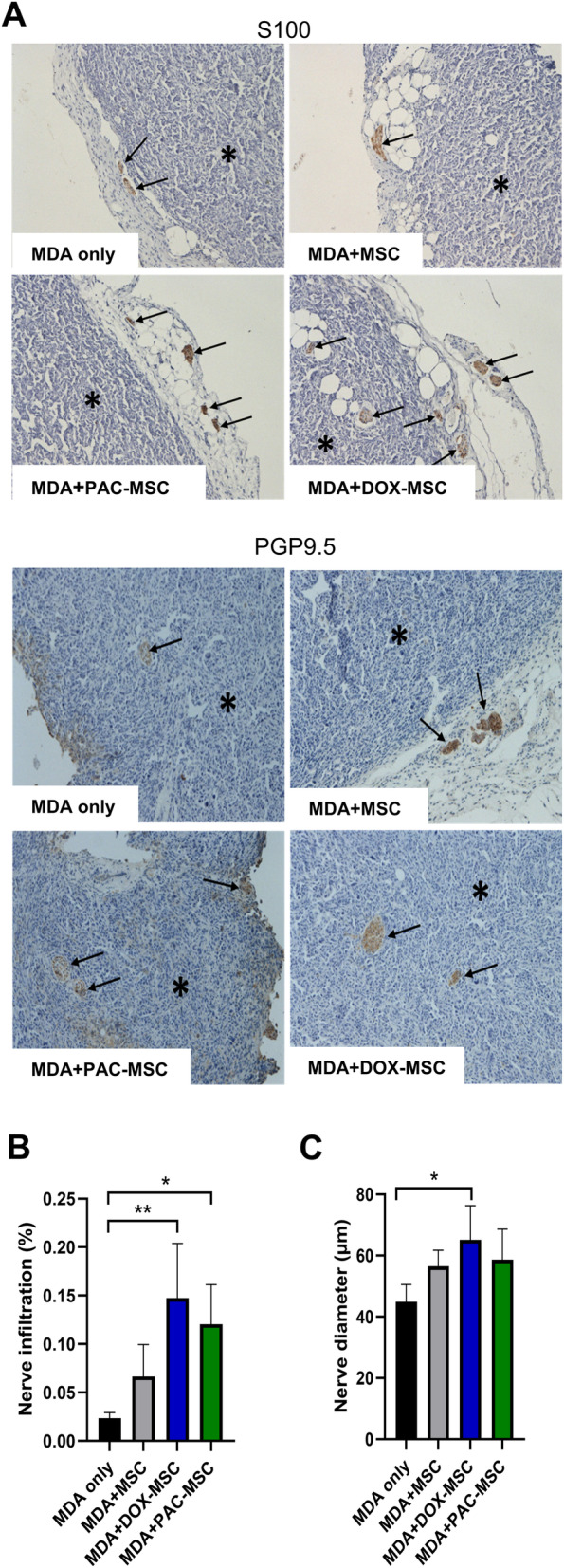
Chemotherapy-exposed MSC and nerve infiltration in tumor xenografts. (**A**) Two different polyclonal antibodies identifying nerve tissue (against S100 protein and PGP9.5 protein) stained nerve structures cross-section area in the tumor (indicated by arrows) and in the adjacent adipose tissue up to 2 mm from the tumor surface. (**B**) Nerve infiltration was expressed as percentage of the tumor section area in each experimental group. Increased nerve infiltration of tumors was observed in co-cultures with DOX-/PAC-MSC. (**C**) DOX-MSC co-injection also increased nerve diameter of detected tumors. For statistical analysis, one-way ANOVA with Tukey’s multiple comparisons test was used. Statistically significant results are highlighted with asterisks at *p* < 0.05 (*), *p* < 0.01 (**)

During the histological analysis of tumor composition, an increased neural infiltration in tumors formed by MDA + DOX-MSC and MDA + PAC-MSC was discovered. The role of the neural system has emerged to be an important modulator in breast cancer progression and metastasis and its contribution in the complex interactions within the tumor microenvironment requires precise consideration. Neural markers S100 and PGP9.5 were used for visualization of nerve fibers in tumor sections (Fig. 4A). The increased innervation was observed in MDA + PAC-MSC and MDA + DOX-MSC tumors (Fig. 4B), where also the nerve diameters were significantly increased compared to MDA-induced tumors (Fig. 4C).

#### Chorioallantoic membrane model

**Fig. 5:**
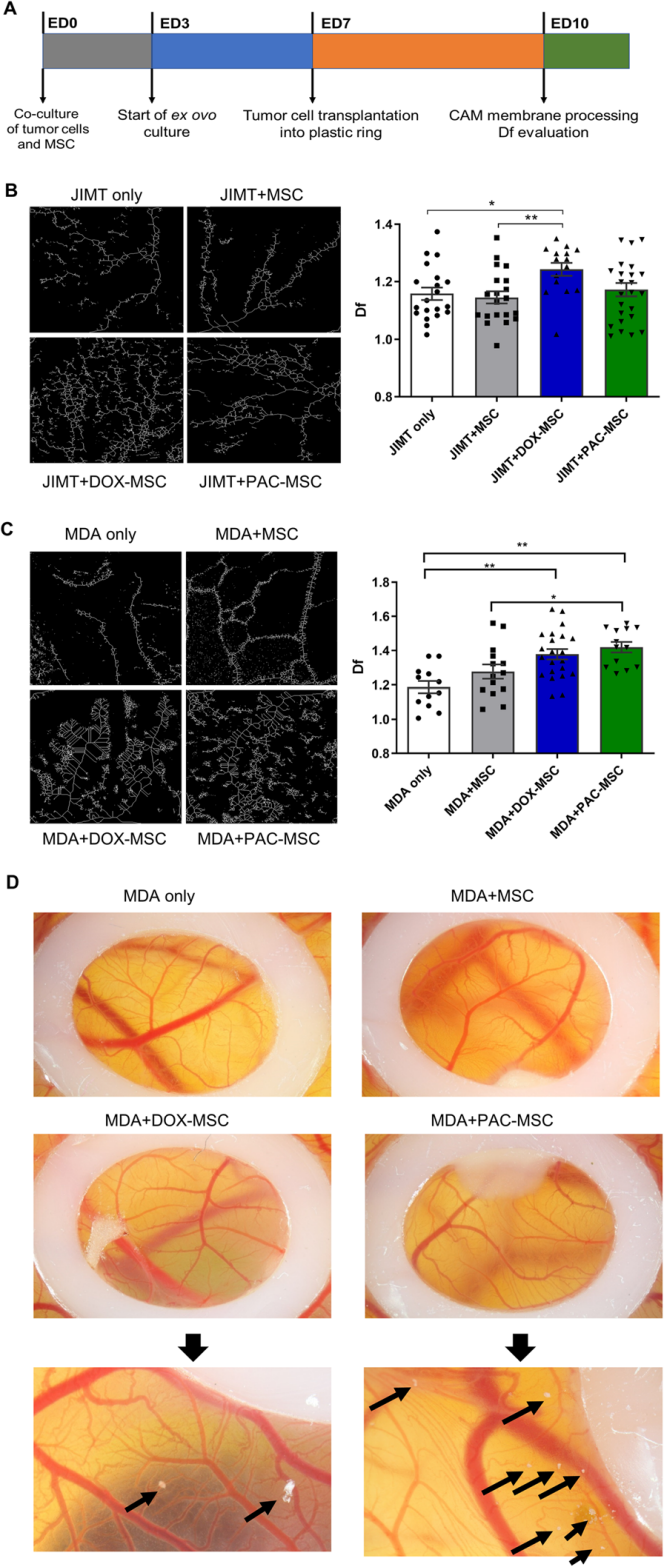
Analysis of angiogenesis in co-cultures of breast cancer cells and chemotherapy-exposed MSC using chorioallantoic model. (**A**) Outline of the experiment – MSC were exposed to the chemotherapy, co-cultured with tumor cells and topically applied on the CAM. (**B**) Evaluation of angiogenesis in co-culture of JIMT + MSC (unexposed or DOX-/PAC-exposed) on CAM by fractal analysis through quantification of fractal dimension (Df). We observed significantly higher vascular growth in DOX-MSC co-culture compared to unexposed MSC co-culture and JIMT only group. (**C**) Fractal analysis was also used to assess the vascularization in MDA co-culture with unexposed and chemotherapy-exposed MSC. Here we observed significantly higher vascular growth in both, DOX- and PAC-MSC co-cultures, compared to MDA only group. When compared to MDA + MSC, PAC-MSC co-culture had significantly more new blood vessels. (**D**) Co-cultures were growing on the CAMs in the area defined by a silicone ring. MDA cells co-cultured with DOX-MSC and PAC-MSC formed micro-metastases (indicated by arrows) outside that silicone ring. One-way ANOVA with Bonferroni multiple comparisons was used for statistical evaluation. Statistically significant results are highlighted with asterisks at *p* < 0.05 (*), *p* < 0.01 (**), *p* < 0.001 (***)

The co-cultures of MDA or JIMT tumor cells with chemotherapy-exposed MSC were topically implanted onto the plastic ring placed on the 7-day old quail embryo and cultivated *ex ovo* for 3 days (following the scheme in Fig. 5A). The angiogenic potential was evaluated by fractal analysis through quantification of fractal dimension (Df) representing the new vascular growth in the extra-embryonic membrane. We have observed significantly higher angiogenic potential in JIMT + DOX-MSC compared to JIMT only (Fig. 5B) and similarly also in triple-negative MDA + DOX-/PAC-MSC-induced CAM models (Fig. 5C). Here, we have detected also metastatic tumor cells migrating outside the plastic ring suggesting the pro-metastatic effect of chemotherapy-exposed MSC on tumor cells (Fig. 5D).

## Discussion

Following chemotherapy, the microenvironment of breast cancer changes the milieu of growth factors. The stromal cells secrete altered levels of cytokines, which favor tumor cells expressing corresponding receptors [10]. A big challenge in breast cancer treatment poses metastases, which are also the main cause of patient death. Recent evidence indicates that the treatment pressure is a major contributor to tumor mutational burden and leads to an increase in the mutation frequency of several known driver genes in metastatic breast cancer [26]. However, breast cancer dissemination is also dependent on the cells in its microenvironment, which are equally affected by the chemotherapy treatment. Karagiannis et al. [27] have shown that chemotherapy (neoadjuvant paclitaxel after doxorubicin plus cyclophosphamide) increases the activity and density of cancer cell invasion sites – so called tumor microenvironment of metastasis (TMEM). They are clinically validated as prognostic markers of metastasis in breast cancer patients suggesting a higher risk of metastatic dissemination. These TMEM sites are composed of three different cell types: tumor cells, perivascular macrophages and endothelial cells, each expressing different proteins influencing tumor cell intravasation and dissemination.

Other cells attracted to tumors are MSC, which represent an important component of TME and were shown to communicate with tumor cells and favor tumorigenesis, migration and chemoresistance [16, 28]. Therefore, we have analyzed how MSC exposure to DOX and PAC affects their phenotype and subsequently leads to distorted communication with tumor cells.

Effects of DOX on MSC have been thoroughly reviewed in a paper by Baxter-Holland and Dass [29]. The adverse effects included lower proliferation rates, induction of apoptosis, reduced differentiation into cardiomyocytes and if induced into cardiomyogenic differentiation, halted expression of cardiac troponin T by MSC. This can partially explain the cardiotoxic effects of DOX. Other adverse effects caused by the anthracycline drug doxorubicin involved cell shrinkage, enhanced ROS production or increased expression of mitogen-activated protein kinases signaling pathway, namely p38 and jun N-terminal kinase. Increased resistance to DOX in triple-negative breast cancer cells which interacted with adipose-derived MSC was evidenced by Chen et al. [30]. The resistance was achieved by increased expression of breast cancer resistance protein BCRP along with increased IL-8 secretion leading to impaired accumulation of DOX within the breast cancer cells [29].

The impact of PAC on MSC’ phenotype and integrity has not been documented in such thorough details. However, Li et al. have evaluated the effect of several chemotherapeutic agents on human MSC derived from bone marrow, and evidenced more than 20 % reduction of viable cell numbers at clinically relevant concentrations. In contrast with other drugs, the MSC sustained suppressed proliferation even after PAC withdrawal, suggested possible induction of apoptosis. Altogether, their data indicate that PAC along with other widely used agents in clinical practice for breast cancer treatment may irreversibly alter the microenvironment in which they reside [31].

The exposure of cells to the chemotherapy can lead to changes in many cellular processes. As published before, MSC react by senescence induction as a response to numerous stress stimuli, including chemotherapeutic agents [32, 33]. We evaluated the induction of senescence after MSC exposure to DOX and PAC and observed partially senescent MSC population after treatment (data not shown). Autophagy shares some common features with senescence. There is evidence that chemotherapy treatment can induce autophagy in cells [34, 35]. In our pilot experiments, we also observed higher autophagy in DOX- and PAC-MSC (data not shown). All of these processes are crucial for the cell homeostasis and targeting them could be a promising therapeutic strategy. Some of the effects described in this paper might be explained by the induction of one or both of these processes as suggested by our preliminary experiments, however, mechanistic modeling requires complex experiments that are beyond the scope of this paper.

In our study, exposure of the MSC to DOX was associated with increased secretion of SDF-1α, IL-32, THBS-1, DPPIV and uPAR when compared to unexposed MSC. Paclitaxel exposure resulted in decreased production of CXCL5, endoglin, Dkk-1 and increased production of IL-32.

The chemotherapy-exposed MSC communicated with triple-negative MDA-MB-231 cells and HER2 + JIMT cells via gap junctions more than unexposed MSC. This could be the reason for higher supportive effect of DOX-MSC and PAC-MSC on the invasive potential of tumor cells *in vivo*, where histological tumor xenograft analysis revealed increased tumor cell invasion only in MDA cells co-injected with DOX-MSC or PAC-MSC. Additionally, tumor invasive front was surrounded by smaller adipocytes with wider intercellular spaces showing rearrangements of the extracellular matrix in close proximity to the invasive front. Those crown-like structures composed of macrophages surrounding dead or dying adipocytes were associated with pro-inflammatory processes by which adipose tissue contributes to a worse prognosis of breast cancer [36] and affects also TME and breast cancer behavior [37]. It was shown, that circulating monocytes or tissue residing macrophages are recruited and stimulated by different tumor-derived signals, such as chemo-attractants, e.g. CCL2 [38, 39] or cytokines, e.g. IL-4, IL-10 and IL-13 [40]. We have observed increased CCL2 secretion in all tumor cell lines co-cultured with chemotherapy-educated MSC (except for MDA + PAC-MSC) but marked difference was apparent mainly after co-culture with MSC exposed to DOX. Moreover, solely in this group, where MDA + DOX-MSC were injected into immunodeficient mice, metastatic tumor cells migrating alongside adipose tissue and the presence of metastatic cells in the lungs were detected. We suppose that also higher levels of SDF-1 and IL-32 produced by MSC after DOX exposure may contribute to the metastasizing into lungs and to the increased angiogenesis via activation of MMPs (reviewed in [41, 42]). The chemokine stroma-derived factor SDF-1 (also called CXCL12) plays multiple roles in tumor pathogenesis. It was shown that in the tumor microenvironment the SDF-1 contributes to tumor vascularization by recruitment of endothelial stem cells [43] and endothelial progenitor cells [44]. Moreover, Muller et al. showed evidence that CXCL12 with its receptor CXCR4 mediates human breast cancer metastasis [45]. Tissue remodeling mostly caused by proteolytic degradation of ECM is a necessary step that allows tumor cells to leave the site of the primary tumor. MMPs are largely involved in the process of ECM degradation. Therefore, activation of these enzymes breaks down the physical barriers and allows the tumor cells to metastasize [46]. Several studies have shown that CXCL12 induced the MMPs synthesis (reviewed in [41]). We also found higher secretion of IL-32 and THBS-1 in the chemotherapy-exposed MSC. IL-32 is also known to be connected to MMPs production [42] and although a precise role of THBS-1 in tumor invasion and migration remains unclear, with compelling evidence suggesting both stimulatory and inhibitory roles (review in [47]). Ndishabandi et al. shown that in breast cancer, higher levels of THBS-1 were found in more migratory breast cancer cells [48]. Additionally, Yee et al. showed that THBS-1 can promote breast cancer to metastasize to the lungs, suggesting that THBS-1 plays a role in mammary cancer cell migration [49]. Karagiannis et al. [27] have associated doxorubicin/cyclophosphamide treatment of transgenic MMTV-PyMT mice bearing spontaneous breast tumors with an increased number of circulating tumor cells as well as increased numbers of perivascular TIE2hi/VEGFhi macrophages compared to vehicle-treated controls. However, we analyzed tumor metastatic gene expression only in the whole tumor mass, where marked changes were not observed, suggesting that analysis of separated cell populations would be more suitable.

On top of increased metastatic potential, also angiogenesis plays an important role in tumor cell dissemination. The co-culture of tumor cells and chemotherapy-exposed MSC with subsequent application on CAM membrane resulted in higher angiogenesis as well as dissemination of tumor cells in case of triple-negative breast cancer cells. An important role in angiogenesis is linked to endoglin, which is required for neo-angiogenesis in tumors, and to the metastatic potential of cancer cells, which is affected by TGFβ receptor signaling modulation [50]. In the MSC context, endoglin is involved in MSC migration to the tumor site [51, 52]. However, our results did not show its changes in DOX-exposed MSC and lower levels in PAC-MSC compared to unexposed MSC, although higher angiogenesis and metastatic potential of cancer cells *in vivo* were associated with exposure to both drugs. Based on these data we assume that various molecules released by chemotherapy-exposed MSC would play their part in the observed effects. When we analyzed the expression of VEGF in co-cultures with DOX-/PAC-MSC, opposite results in VEGF expression were achieved (increased expression of VEGF in case of JIMT cells and decreased in MDA cells, data not shown). This suggests that even though chemotherapy-exposed MSC caused a similar effect on angiogenesis, the underlying mechanism might differ for individual molecular subtypes. According to Zhao et al. [53], breast adipose tissue-derived MSC can promote angiogenesis by more than one mechanism. They are capable to assemble into vessel-like structures and promote angiogenesis by different mechanisms, which could partially explain why we observed different VEGF expression although the angiogenesis was increased in both cell lines. To decipher our findings in the clinical context, we cannot forget patient-specific differences.

In addition to the higher invasive potential of tumor cells co-injected with chemotherapy-exposed MSC, we have observed also increased neural infiltration and increased nerve fibers formation. The sympathetic nervous system, and mainly the adrenergic signaling, was found to be correlated with poor prognosis and metastatic rate in breast cancer [13, 54, 55]. Di Huang et al. [56] demonstrated that the thickness of tumor-infiltrating nerve fibers was associated with shorter disease-free survival, poor differentiation, lymph node metastasis, high clinical staging, and triple-negative subtype in breast cancer. On the other hand, the reservations about systemic administration of β-AR antagonists which can have unpredictable consequences on the progression of breast cancer were also discussed [15]. The exact molecular mechanism responsible for chemotherapy-induced effects needs to be properly examined.

## Conclusions

Our data suggest that neoadjuvant chemotherapy could alter otherwise healthy stroma in breast tissue into misled tumor-promoting and metastasis favoring hostage. Factors from the TME can modify the therapeutic effect of the chemotherapy, facilitating the escape and survival of clonal cell populations. Targeting the tumor microenvironment and its complex net of signals, therefore, raises hope that the standard therapy might not fail in the end, but accomplish the curative purpose.

## Supplementary Information

**Additional file 1.**

## Data Availability

All the data that support this study are presented in the paper. Any reasonable requests for additional evidence should be directed to corresponding author.

## Abbreviations

BC: Breast cancer
BCC: Breast cancer cells
CAM: Chorioallantoic membrane
CHAE: Chloracetate esterase
DOX: Doxorubicin
DOX-MSC: Doxorubicin-exposed mesenchymal stromal cells
Df: Fractal dimension
ECM: Extracellular matrix
EMT: Epithelial-to-mesenchymal transition
GJIC: Gap-junctional intercellular communication
MSC: Mesenchymal stromal cells
PAC: Paclitaxel
PAC-MSC: Paclitaxel-exposed mesenchymal stromal cells
TME: Tumor microenvironment
TMEM: Tumor microenvironment of metastasis
VIM: Vimentin
