# Supplementary material for: Chemotherapy-triggered changes in stromal compartment drive tumor invasiveness and progression of breast cancer
Source: J Exp Clin Cancer Res. 2021 Sep 27;40:302. doi: 10.1186/s13046-021-02087-2 (PMC8477536; doi:10.1186/s13046-021-02087-2)
Supplement: Supplementary file 1 — Additional file 1. [file 13046_2021_2087_MOESM1_ESM.docx]

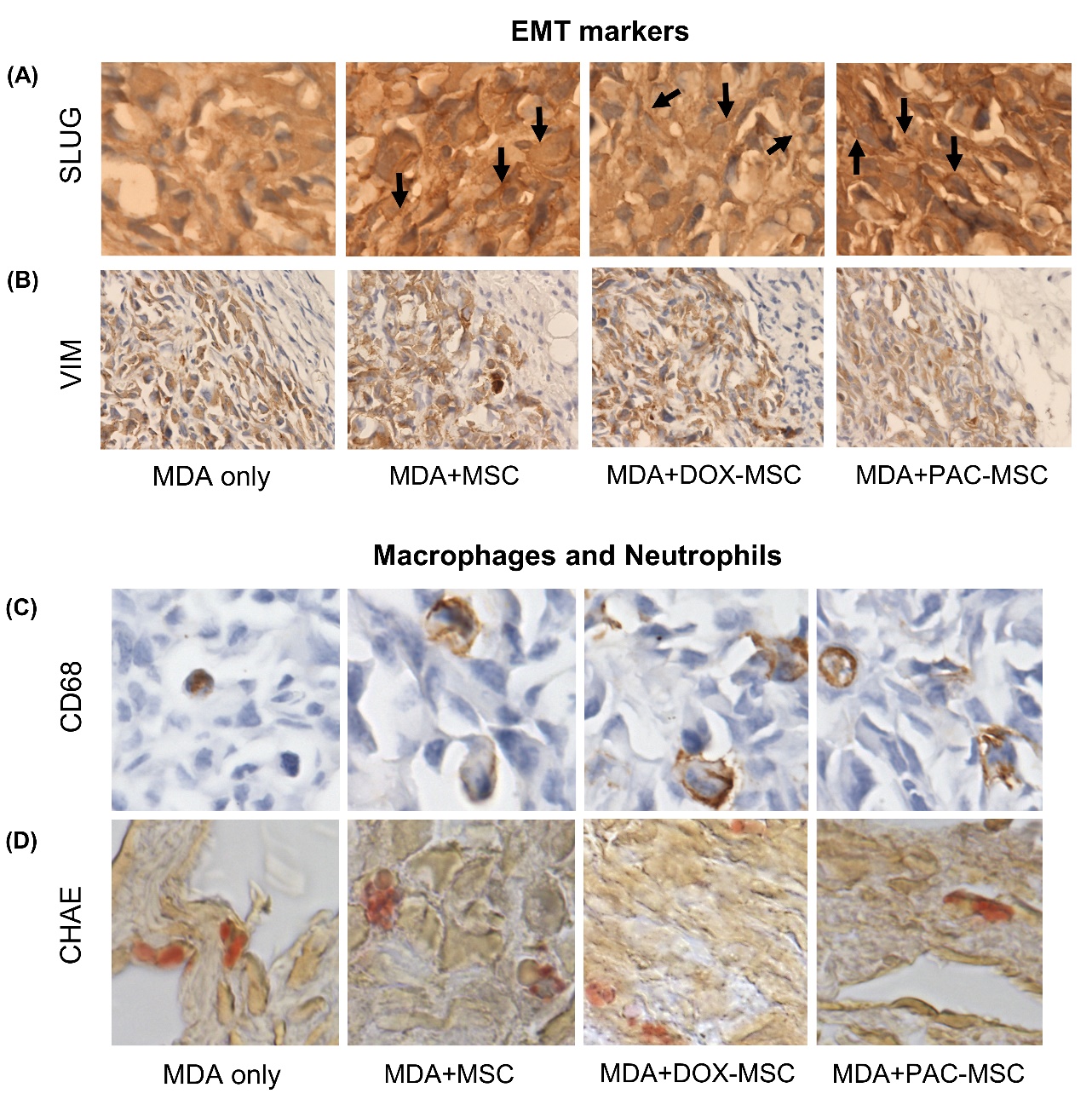


**Additional file 1: Immunohistochemical detection of epithelial-to-mesenchymal transition and the presence of macrophages and neutrophils in mice xenografts.** (A) IHC detection of SLUG protein expression - differences between the groups are not prominent, expression in tumors without accompanying MSC is slightly lower when compared to tumors implanted with MSC. There is a tendency to increased SLUG protein presentation on tumor cells membrane (arrows), not significantly affected by chemotherapeutic treatment (DOX or PAC). Immunoperoxidase technique, diaminobenzidine, 400x. (B) IHC detection of vimentin expression - no apparent differences between the groups were noted. Immunoperoxidase technique, diaminobenzidine, 200x. (C) Detection of macrophages using the anti-CD68 antibody and (D) neutrophilic granulocytes using CHAE enzyme activity - macrophages and neutrophils were only scarce (< 2/2mm^2^) in MDA only group, injection of MDA with MSC lead to 10-fold increase of neutrophils, mostly at the periphery of the tumor. Same effect was observed on the content of macrophages and with further increase in density of macrophages infiltrate in tumors administered with MSC exposed to DOX or PAC. CD68 – immunoperoxidase, diaminobenzidine; CHAE - α-naphtol-AS-D-chloroacetate; 600x.
